# Exogenous silicon enhances cadmium-stressed cucumber seedling establishment by modulating antioxidant and hormonal pathways

**DOI:** 10.3389/fpls.2026.1851047

**Published:** 2026-07-13

**Authors:** Lijun Zhu, Fengli Yang, Yanping Mao, Hongying Xie, Baoguo Du

**Affiliations:** 1 School of Life Sciences (School of Ecological Forestry), Mianyang Teachers’ College, Mianyang, China; 2 Forest Ecology and Conservation in the Upper Reaches of the Yangtze River Key Laboratory of Sichuan Province, Mianyang, China; 3 Sichuan Jiuzhai Valley National Ecological Quality Comprehensive Monitoring Station, Mianyang, China; 4 Key Laboratory of Research and Conservation of Biological Diversity in Minshan Mountain of National Park of Giant Pandas at Mianyang Teachers’ College of Sichuan Provincial Department of Education, Mianyang, China

**Keywords:** abscisic acid, antioxidant system, cucumber, gibberellin, heavy metal contamination, hormone regulation, seed germination, silicon

## Abstract

It has been demonstrated that silicon (Si) has the capacity to enhance the tolerance of plants to abiotic stress; however, there is a paucity of research regarding its function in alleviating heavy metal toxicity during the processes of seed germination and early seedling growth. In the present study, we firstly examined the effects of Si on the germination and early growth of cucumber (*Cucumis sativus* L.) seeds under Cd stress, and further investigated the biochemical mechanisms. The results demonstrated that 2 mM Si significantly alleviated Cd-induced inhibition of radicle elongation and growth. Adding Si resulted in a reduction in H_2_O_2_ accumulation, an increase in the soluble sugar content, and elevated antioxidant enzyme (SOD, POD, CAT) activities and their related genes (*CsSOD_(Cu-Zn)_*, *CsPOD-2–4* and *CsCAT*) expression levels. Application of Si also led to a decrease in ABA concentration and an increase in total GA content. The analysis revealed that the genes involved in ABA biosynthesis (*CsNCED1*, *CsNCED2*) were downregulated concurrently with the upregulation of a gene involved in ABA catabolism (*CsCYP707A1*). Concurrently, the expression of GA content was enhanced, concomitant with the upregulation of *CsGA_20_OX*, *CsGA_3_OX_2_* and *CsGA_3_OX_3_*. The alleviation mechanism involved in mediating redox and hormonal status was tested by adding ABA, GA biosynthesis and H_2_O_2_ scavenging inhibitors. The findings of this study highlight the potential of eco-friendly Si in alleviating Cd-induced inhibition of post-germinative growth and seedling establishment against heavy metal contamination, and the pivotal role of redox and hormonal status involved in seed germination.

## 1 Introduction

The widespread presence of heavy metals in agricultural soils, primarily as a result of industrial activities, urbanization and the excessive use of agrochemicals, is a growing concern (Coskun et al., 2025). Among the various heavy metal pollutants in China, cadmium (Cd) is considered the most prominent pollutant, with a national exceedance rate of 7% (Zhao et al., 2015). Which inhibits seed germination and disrupts plant growth through various physiological impairments such as oxidative stress and cellular homeostasis disruption (Hu et al., 2025).

Silicon (Si) is the second most abundant element in the Earth’s crust after oxygen (Al Murad et al., 2020). It has been widely recognized for its beneficial role in enhancing plant growth and development under both various stress conditions (Etesami and Jeong, 2018; Arif et al., 2021). Many studies have primarily focused on several key protective mechanisms, e.g., mitigating oxidative damage (Kim et al., 2017; Farouk and Al-Sanoussi, 2019), alleviating nutrient imbalance-induced stress to promote growth, development and yield (Fatemi et al., 2021), improving leaf water status and photosynthetic efficiency (Souri et al., 2021), and reducing cinnamic acid-induced autotoxicity stress (Lyu et al., 2022). However, relatively few studies have reported on the potential of Si on alleviating the detrimental effects of heavy metal stress, particularly during seed germination and early seedling growth.

Seed germination represents the most critical and sensitive phase in early plant development, as it underpins seedling establishment, morphology, and ultimately crop yield (Jatana et al., 2024). This process is highly impacted by a variety of biotic and abiotic factors (Byregowda et al., 2024). For example, hydrogen peroxide (H_2_O_2_) is a key signaling molecule with dual effects on germination and seedling growth, depending on its concentration (Wang et al., 2025a). Low to moderate H_2_O_2_ promotes germination by activating antioxidant defenses and cell wall loosening, while excessive H_2_O_2_ causes oxidative stress and inhibits radicle emergence (Jira-Anunkul and Pattanagul, 2020). Soluble sugars provide not only carbon skeletons and compatible solutes for the growing embryo axis energy, some small-molecule sugars (e.g., glucose, sucrose) act as signaling molecules that regulate gene expression and interact with the abscisic acid (ABA)/gibberellins (GA) pathway (Bozdar et al., 2025; Byregowda et al., 2024). The antagonistic balance between ABA and GA acts as the central hormonal switch, i.e., ABA promotes dormancy and inhibits germination, whereas GA break ABA-triggered seed dormancy (Luo et al., 2024; Iradukunda et al., 2024; Xia et al., 2023). However, the extent to which exogenous application of beneficial elements modulates the redox pathways and hormonal status induced by heavy metal stress remains to be fully elucidated.

In order to explore the potential of Si in alleviating Cd-induced inhibition of seed germination and early seedling growth under Cd stress and reveal its mechanism, four experiments were conducted using cucumber (*Cucumis sativus* L.) seeds. Initially, the impacts of varying Cd concentrations on seed germination and early seedling growth were examined, and a suitable Cd concentration was identified for subsequent experimentation. Secondly, the study examined the varying levels of Si in relation to the alleviation of the declined seed germination and growth induced by Cd treatment. Thirdly, the biochemical mechanisms underlying Si-mediated protection were investigated, focusing on oxidative stress, antioxidant defense, hormone regulation (ABA and GA), as well as the gene expression levels. Finally, ABA and GA inhibitors and a H_2_O_2_ scavenger were used to test the mechanism by which Si alleviates suppressed seed germination under Cd stress. Cucumber was selected as the model crop because it is widely cultivated and China accounts for about 77% of global cucumber production, and it is very sensitive to heavy metal stress (Li et al., 2017; Azarmi and Hosseini, 2022; Meng et al., 2024).

## 2 Materials and methods

### 2.1 Seeds and preparation

**C**ucumber (*Cucumis sativus* cv. Jinrui 100) seeds were acquired from the Sichuan Academy of Agricultural Science in Chengdu, China. Healthy seeds were surface-sterilized by soaking in 0.1% (v/v) NaClO for 10 min and then rinsed six times with distilled water before subjected to germination.

### 2.2 Experimental design

#### 2.2.1 Effects of different Cd concentrations on seed germination and growth

To investigate the impact of different Cd concentrations on cucumber seed germination and early seedling development, a set of seven concentrations, i.e., 50, 100, 150, 200, 250, 300, and 350 mg/L of CdCl_2_ (Sigma, Shanghai, China) dissolved in distilled water were applied. An extra treatment with only distilled water was employed as control. For each treatment, 50 sterilized seeds were arranged in 9 cm Petri dishes lined with double layers of filter paper, and 10 mL of the corresponding treatment solution (distilled water for control) was added to each dish. A total of 10 dishes (500 seeds per treatment) were incubated in a growth chamber (LGZ-260Y, Green Bo Instrument Co., Ltd, Hangzhou, China) with a 16-hour photoperiod at a photosynthetic photon flux density (PPFD) of 100 μmol·m^-^²·s^-^¹ and 25 ± 1 °C for 7 d. The filter papers and the solutions were replaced by new ones every three days to maintain moisture and Cd concentrations. Germination was recorded when the radicle visibly penetrated the seed coat. On the 7th day, the primary root length and the fresh weight of roots were measured across all treatments. Based on the significant reductions both in primary root length and root fresh weight, 150 mg/L Cd was used for subsequent experiments. Although root length and fresh weight further declined with increasing Cd concentrations, the Cd≥200 mg/L is far in excess of the values commonly reported in agricultural soils. It is hypothesized that this sub-lethal concentration may facilitate a reliable assessment of Si-mediated alleviation, as has been adopted in other studies (Wang et al., 2025a).

#### 2.2.2 Effects of Si on seed germination and early seedling growth under Cd Stress

To evaluate the effects of Si addition, sterilized seeds were placed in petri dishes containing 150 mg/L Cd solution and different concentrations of sodium silicate (Sigma), i.e., either 0, 1, 2, 3, 4, and 5 mM Na_2_SiO_3_. The total volume in each petri dish was 10 mL. Ten Petri dishes with 500 seeds per treatment (50 per dish) were incubated under the same conditions as described in section 2.2.1. On the 7th day, primary root length and the fresh weights of hypocotyl-cotyledon and root were measured. The concentration of 2 mM Si was found to be optimal for alleviating Cd-induced inhibition and was used in subsequent experiments.

#### 2.2.3 Biochemical analysis of germinated seeds under Cd and Si treatments

Surface-sterilized seeds (10 petri dishes per treatment, 50 seeds per petri dish) were exposed to one of the following treatments: Cd treatment (150 mg/L Cd, 10 mL), Si+Cd (150 mg/L Cd + 2 mM Si, 10 mL), and control (only 10 mL distilled water without Cd and Si). Germination and growth procedures followed the same protocol as in section 2.2.1. A total of 6 replicates (approximately 100 mg germinated seeds) were collected from the 10 petri dishes of each treatment at day 3, 4 and 5, respectively. Contents of soluble sugar, H_2_O_2_, ABA and GA, the activities of antioxidant enzymes (SOD, POD, CAT), and transcript levels of genes associated with antioxidant enzymes and hormones were analyzed.

#### 2.2.4 Verification with hormone inhibitors and H_2_O_2_ scavenger

An experiment was conducted to verify the hypothesis that Si alleviates seed germination under Cd stress through the mediation of hormonal and redox status. For this purpose, surface-sterilized seeds were germinated in a solution containing 150 mg/L Cd, along with either 100 μM of the ABA biosynthesis inhibitor nordihydroguaiaretic acid (NDGA, Sigma; Han et al., 2004), or 10 μM of the GA biosynthesis inhibitor paclobutrazol (PCB, Sigma; Nicolás et al., 1996), with or without supplementation of 2 mM Si. In a parallel experiment, seeds were exposed to a 150 mg/L Cd solution containing 5 mM of the H_2_O_2_ scavenger dimethylthiourea (DMTU, Sigma; Liu et al., 2015), with or without 2 mM Si. A control group was set up using distilled water. Ten petri dishes consisting of 500 seeds per treatment were incubated as previously described. Primary root lengths of seed from each treatment were measured after seven days.

### 2.3 Biochemical analysis

#### 2.3.1 Determination of H_2_O_2_ content

The extraction and quantification of H_2_O_2_ were performed according to the method of Velikova et al. (2000). Briefly, 200 mg of germinated seeds from each treatment were homogenized in 2 mL of ice-cold 0.1% (w/v) trichloroacetic acid (TCA). The homogenate was centrifuged at 12,000 g for 20 min. Subsequently, 0.5 mL of the resulting supernatant was combined with 1.5 mL of a phosphate buffer solution (pH 7.0) containing 1 M potassium iodide (KI) and 10 mM potassium phosphate. The absorbance of the mixture was recorded at 390 nm using a V-1100 spectrophotometer (Shanghai Mei Analytical Instruments Co., Ltd.). The H_2_O_2_ concentrations were then determined based on a pre-established standard curve.

#### 2.3.2 Determination of soluble sugar

Soluble sugar contents were quantified following a method of Yemm and Willis (1954). For this purpose, 100 mg homogenized seeds were extracted with 70% (v/v) ethanol before being dried under vacuum conditions. The dried extracts were dissolved in warm water and cleared with aluminum hydroxide. Five mL anthrone reagent (100 mg anthrone in 50 mL 70% H_2_SO_4_) was then added in the extract solution, and then the mixture was heated in boiling water bath for 15 min. The absorbance of the cooling mixture was read at 625 nm using a spectrophotometer (V-1100, Shanghai Mei Analytical Instruments Co., Ltd, Shanghai, China). Soluble sugar contents were calculated according to a standard curve subjected to the same manner.

#### 2.3.3 Determination of antioxidant enzyme activities

The enzyme activities were determined through the homogenization of 200 mg of germinated seeds with 3 mL of ice-cold HEPES buffer (25 mM, pH 7.8) containing 0.2 mM EDTA, 2 mM ascorbic acid, and 2% Polyvinyl pyrrolidone. The homogenates were subjected to a centrifugal process at 12,000 g, at a temperature of 4 °C for a duration of 20 minutes. Thereafter, the resulting supernatant was utilized for the assessment of enzymatic activity (Wang et al., 2011). The activity of superoxide dismutase (SOD) was quantified by measuring the inhibition of nitro blue tetrazolium (NBT) chloride photochemical reduction at 560 nm (Stewart and Bewley, 1980). The activity of peroxidase (POD) was quantified by monitoring the increase in absorbance at 470 nm due to guaiacol oxidation (Zhu et al., 2013). The activity of catalase (CAT) was determined by the absorbance decrease at 240 nm due to the H_2_O_2_ decomposition (Dong et al., 2014). The absorbance was measured with a spectrophotometer (V-1100, Shanghai Mei Analytical Instruments Co., Ltd.) at 25 °C.

#### 2.3.4 Determination ABA and GA contents

Approximately 100 mg of seed material was homogenized in liquid nitrogen. ABA was extracted in 10 mL of 80% methanol containing 40 mg L^-^¹ butylhydroxytoluene for 24 h at 4 °C in darkness. GA mixture was extracted in 100 mL of 80% (v/v) methanol for 12 h at 4 °C. After centrifugation at 10,000 g for 20 min at 4 °C, the supernatant was collected (Julliard et al., 1994). Sample purification was carried out by solid-phase extraction (SPE) on Oasis MCX cartridges (mixed-mode, reversed-phase/cation-exchange) followed by HPLC fractionation, according to Dobrev and Kamínek (2002). The purified fractions were then quantified using competitive ELISA kits (Enzyme-linked Biotechnology Co., Ltd., Shanghai, China). All assays were performed following the manufacturers’ instructions. The GA contents determined in the present study represent the contents of total gibberellins.

#### 2.3.5 Total RNA extraction and real-time quantitative PCR analysis

The transcript abundance of genes associated with antioxidant enzymes and hormones was analyzed in germinated seeds collected on day 3, 4 and 5, respectively. Gene-specific primers were designed based on peptide sequences from mass spectrometry analysis (Xu et al., 2012; Zhang et al., 2014; Zhu et al., 2021) using Primer Premier 5 software, with reference to the NCBI (http://www.ncbi.nlm.nih.gov) and cucumber genomics databases (http://www.cucumberdb.com/). The primers used in this study were listed in **Supplementary Table 1**. Total RNA was isolated from germinated seeds using Trizol Reagent (Biosharp, China) according to the manufacturer’s instructions. The first-strand cDNA was generated by MonScript™ RTШ Super Mix with dsDNase (Two-step) kit (Monad, China), as directed by the guidebook. Subsequently, the qPCR assay was conducted by MonAmpTM SYBR Green qPCR Mix (None ROX) kit (Monad, China) and the reaction was carried out on qTOWER Real-time fluorescence quantitative PCR instrument. *Cs*Actin was used as the reference gene (Zhang et al., 2014). The conditions for qPCR were as follows: The initial temperature of 95 °C was maintained for a duration of 3 min. This was followed by 40 cycles of 10 s at 95 °C and 45 s at 58 °C. RNA purity was assessed using a NanoDrop spectrophotometer (A260/A280 ratio between 1.8 and 2.0). Primer efficiency was determined from standard curves generated by serial 10-fold dilutions of cDNA; all primers exhibited efficiencies between 95% and 105% (R² > 0.99). Melting curve analysis (60–95 °C) was performed after each qPCR run to confirm the amplification of a single specific product (Bustin et al., 2009; Livak and Schmittgen, 2001). For qRT-PCR, six independent biological replicates were performed; for each biological replicate, three technical replicates were run, and the mean Ct value was used for relative quantification according to the method of Livak and Schmittgen (2001). To obtain a ΔCt value, the threshold cycle (*C*t) value of actin was subtracted from that value of the gene of interest. All primer sequences are listed in **Supplementary Table 1**. The analyzed genes included: *CsSOD_(Cu-Zn)_*, *CsPOD-2-4*, *CsCAT* (antioxidant enzymes); *CsNCED1*, *CsNCED2* (ABA biosynthesis), *CsCYP707A1* (ABA catabo- lism) and *CsGA_20_OX*, *CsGA_3_OX_2_*, *CsGA_3_OX_3_* (GA biosynthesis).

### 2.4 Statistical analysis

Morphological data were obtained from 10 independent biological replicates. Biochemical data were obtained from 6 independent biological replicates. One-way analysis of variance (ANOVA) with Duncan’s multiple range test for *post-hoc* comparisons was used to test the differences between the treatments within each parameter. Two-way ANOVA was employed to assess the differences between treatment, time, and their interaction. Statistical analyses and figure generation were performed using SPSS 22.0 (International Business Machines China Co., Ltd., Chengdu, China).

## 3 Results

### 3.1 Effects of Cd on germination and seedling morphology

**Figure 1:**
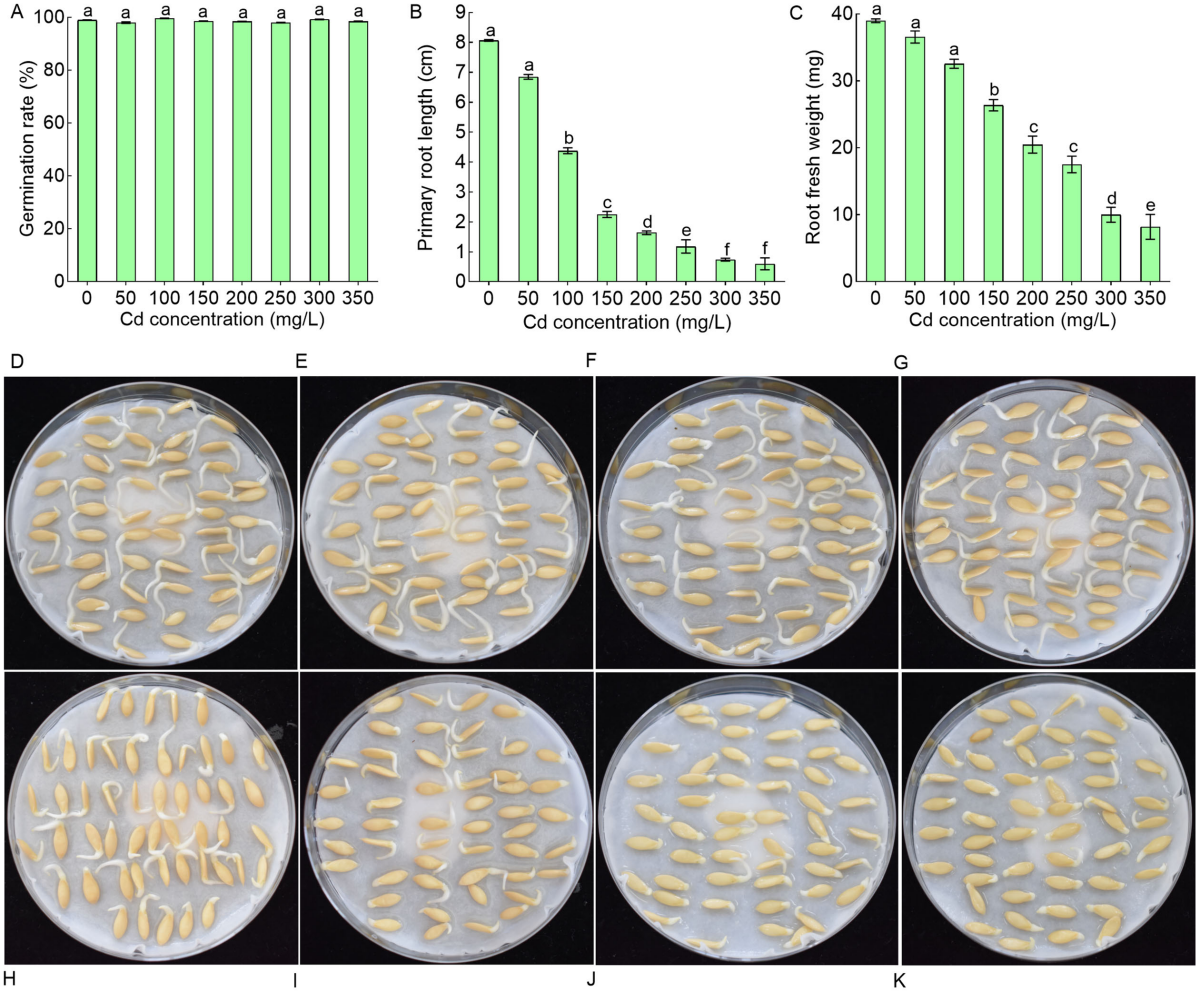
Effects of Cd treatments on germination rate **(A)**, primary root length **(B)** and root fresh weight **(C)** of cucumber seeds determined at day 7. Images **(D–K)** show germination status at day 3 under 0 **(D)**, 50 **(E)**, 100 **(F)**, 150 **(G)**, 200 **(H)**, 250 **(I)**, 300 **(J)**, and 350 **(K)** mg/L Cd treatment. Data are presented as mean ± SE (n = 10). Significant *(p < 0.05*) differences are denoted by different lowercase letters.

Compared to the control group, Cd treatments had no significant effect on the germination rate but did have strong effects on the radicle trait (**Figure 1**). With increasing Cd concentrations, both the primary root length and root fresh weight gradually decreased. At 150 mg/L Cd, highly significant differences (*p* < 0.01) were observed in primary root length, and significant differences (*p* < 0.05) in root fresh weight (**Figures 1B, C**), which resulted in declines of 72.11% and 19.59% for primary root length and root fresh weight, respectively. Therefore, Cd treatment with a concentration of 150 mg/L was selected for subsequent experiments.

### 3.2 Effects of Si on radicle elongation and biomass accumulation under Cd stress

**Figure 2:**
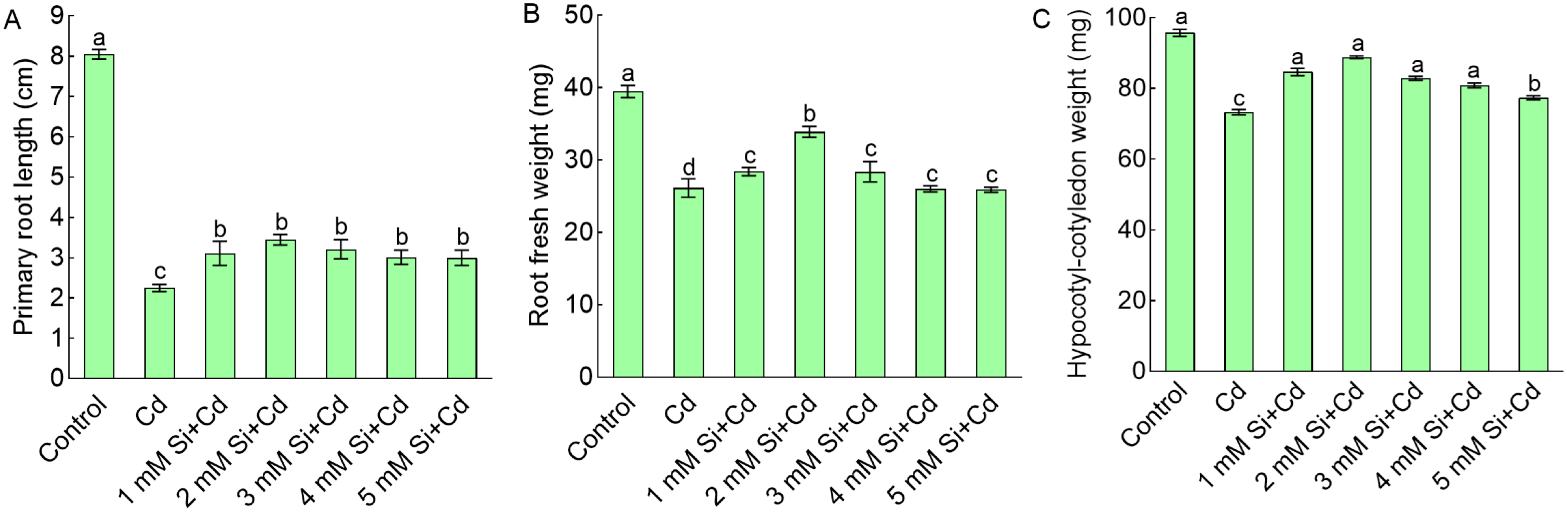
Primary root length, root fresh weight and hypocotyl-cotyledon weight of cucumber seeds under Cd stress with Si addition at day 7. Treatments: CK (control); 150 mg/L Cd + 1–5 mM Si. **(A)** Primary root length; **(B)** Root fresh weight; **(C)** Hypocotyl-cotyledon weight. Data are presented as mean ± SE (n = 10). Significant (*p < 0.05*) differences are denoted by different lowercase letters.

Application of Si (from 1 mM) significantly alleviated the suppressive effects of Cd on primary length, and the fresh weights of hypocotyl-cotyledon and root (**Figure 2**). At a Si concentration of 2 mM, the radicle length, root fresh weight, and hypocotyl-cotyledon fresh weight all reached the highest levels. Higher than 2 mM Si did not show further improvement on germination trait and the early-stage cucumber seedlings. Consequently, a concentration of 2 mM Si was selected for subsequent experiments investigating the physiological mechanisms.

### 3.3 Effects of Si on H_2_O_2_ and soluble sugar contents under Cd Stress

**Figure 3:**
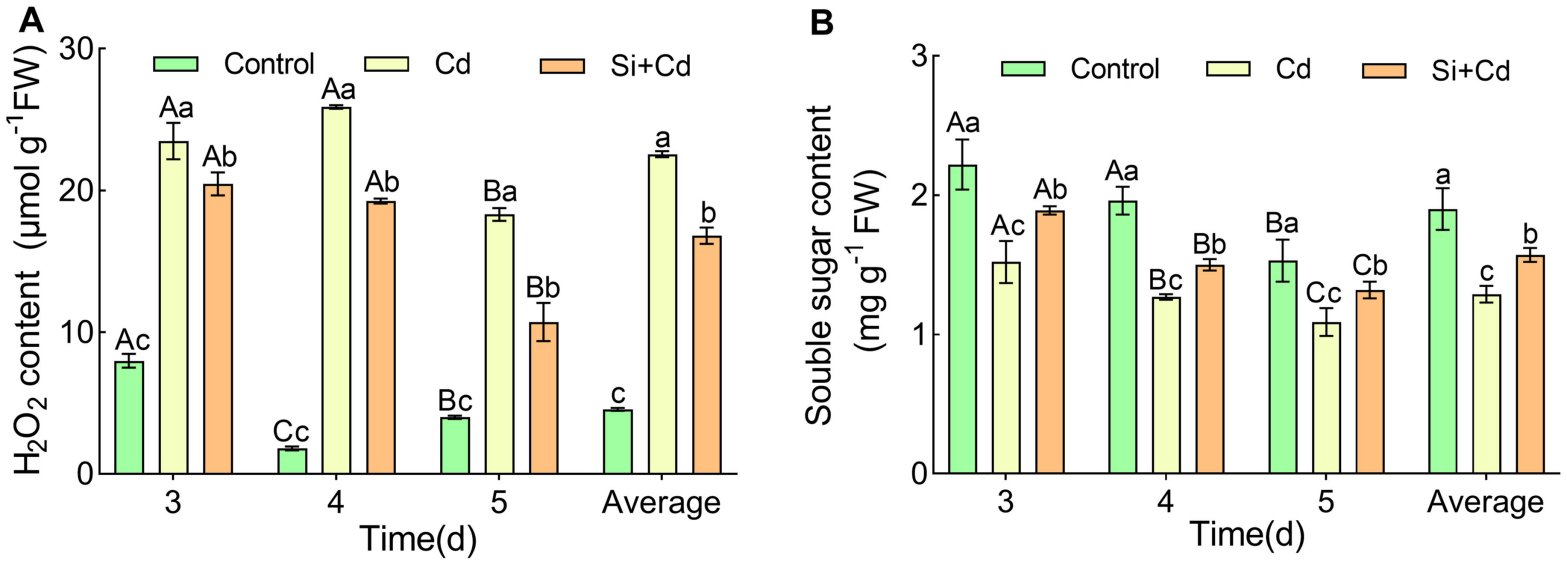
H_2_O_2_ contents **(A)** and Soluble sugar **(B)** in cucumber radicles under control, Cd (150 mg/L Cd) and Cd + Si (2 mM Si + 150 mg/L Cd) treatments at 3, 4, 5 d and the average of these three days (Average). The Data are presented as mean ± SE (n = 6). Different uppercase letters indicate significant differences among time points within the same treatment. Different lowercase letters indicate significant differences among treatments at the same time point (*p* < 0.05). FW, fresh weight.

Compared to controls, Cd treatment resulted in dramatically higher H_2_O_2_ concentrations from day 3 to day 5. Addition of Si alleviated the increased H_2_O_2_ concentrations caused by Cd treatment, but still significantly higher than controls (**Figure 3A**). Cd treatment resulted in significantly decreased soluble sugar contents compared to controls, and the addition of Si improved the soluble sugar contents, but still less than the control levels (**Figure 3B**). Two-way ANOVA analysis revealed that the contents of H_2_O_2_ and soluble sugar within the control and the same treatment group varied across cultivation time. Furthermore, a significant treatment × time interaction for H_2_O_2_ contents was also observed (**Supplementary Table 1**).

### 3.4 Effects of Si on antioxidant enzyme activities under Cd stress

**Figure 4:**
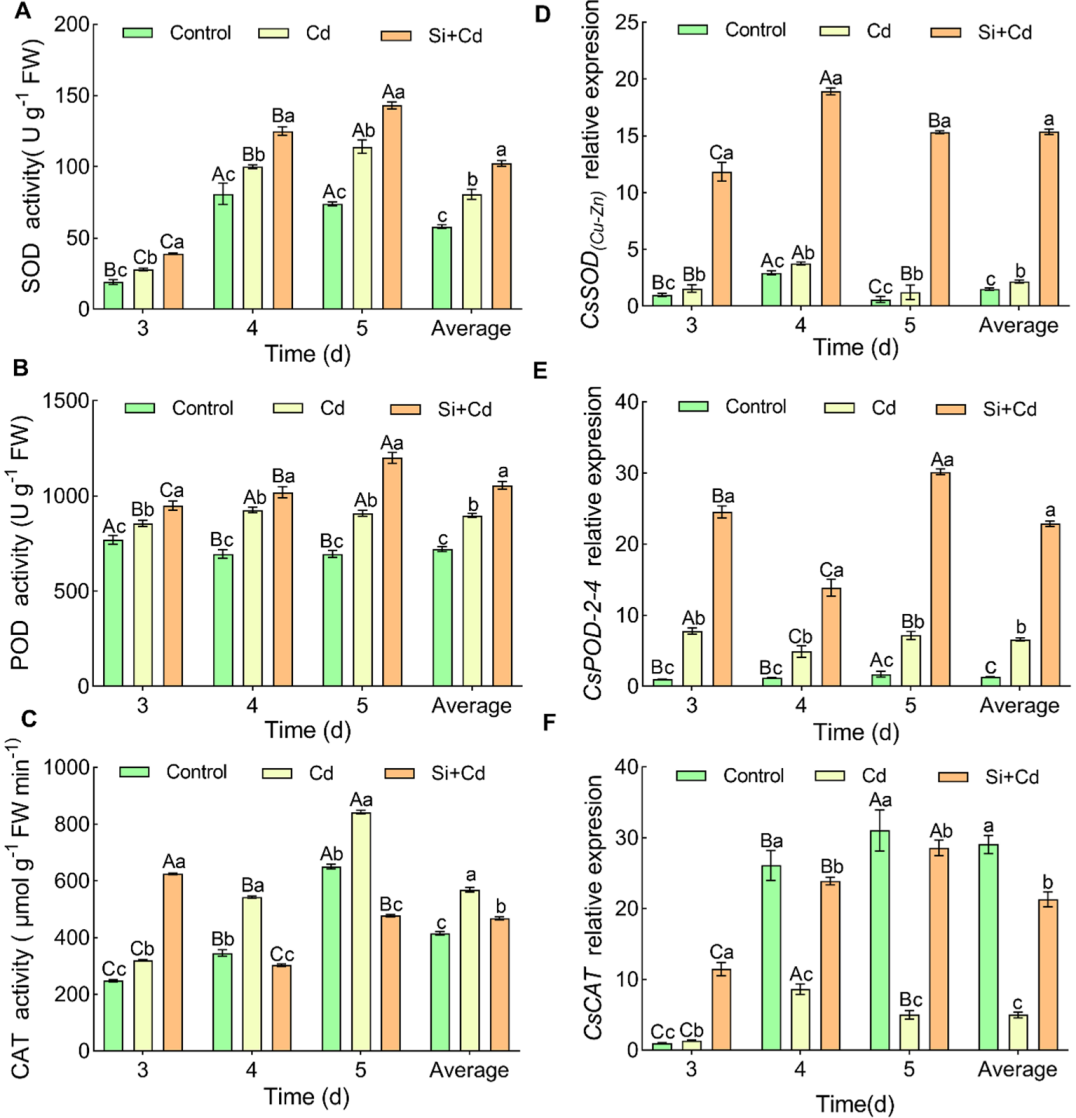
Activities of SOD **(A)**, POD **(B)**, and CAT **(C)**, and transcript levels of *CsSOD_(Cu–Zn)_* **(D)**, *CsPOD-2–4* **(E)**, and *CsCAT* **(F)** in cucumber seeds under control, Cd (150 mg/L Cd) and Cd + Si (2 mM Si + 150 mg/L Cd) treatments at 3, 4, 5 d and the average of these three days (Average). The Data are presented as mean ± SE (n = 6). Different uppercase letters indicate significant differences among time points within the same treatment. Different lowercase letters indicate significant differences among treatments at the same time point (*p* < 0.05). FW, fresh weight.

Compared to controls, Cd treatment caused significantly higher activities of SOD, POD and CAT as well as the transcription levels of *CsSOD_(Cu-Zn)_*, *CsPOD-2-4*, and *CsCAT* with the exception at day 4 and 5. Addition of Si further increased the activities of these enzymes and the transcription levels (**Figure 4**). Enzyme activities in the controls and the two treatments exhibited an overall increase in conjunction with cultivation time, with the exception of CAT activities in the Si+Cd treatment, which demonstrated a decline after day 3. Two-way ANOVA analysis revealed a significant treatment × time interaction for POD and CAT activities, as well as for transcript levels of *CsSOD(Cu-Zn)*, *CsPOD-2-4*, and *CsCAT*. No significant interaction was detected for SOD activity (**Supplementary Table 2**). As shown in **Figure 4**,

### 3.5 Effects of Si on ABA and GA contents and the relative expression levels of related genes under Cd stress

**Figure 5:**
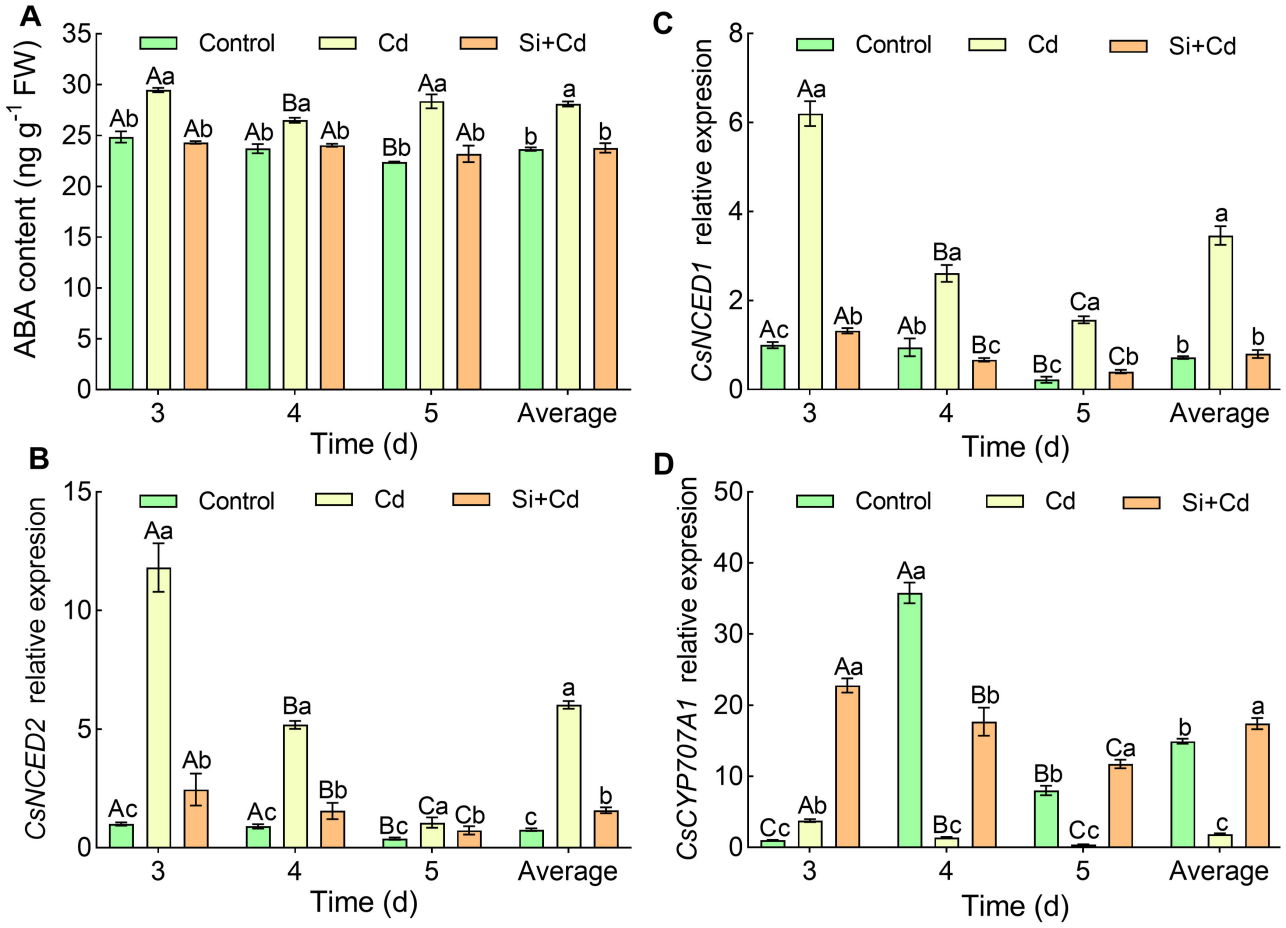
ABA content **(A)**, transcript levels of *CsNCED2* **(B)**, *CsNCED1* **(C)**, and *CsCYP707A1* **(D)** in cucumber seeds under control, Cd (150 mg/L Cd) and Cd + Si (2 mM Si + 150 mg/L Cd) treatments at 3, 4, 5 d and the average of these three days (Average). The Data are presented as mean ± SE (n = 6). Different uppercase letters indicate significant differences among time points within the same treatment. Different lowercase letters indicate significant differences among treatments at the same time point (*p* < 0.05). FW, fresh weight.

**Figure 6:**
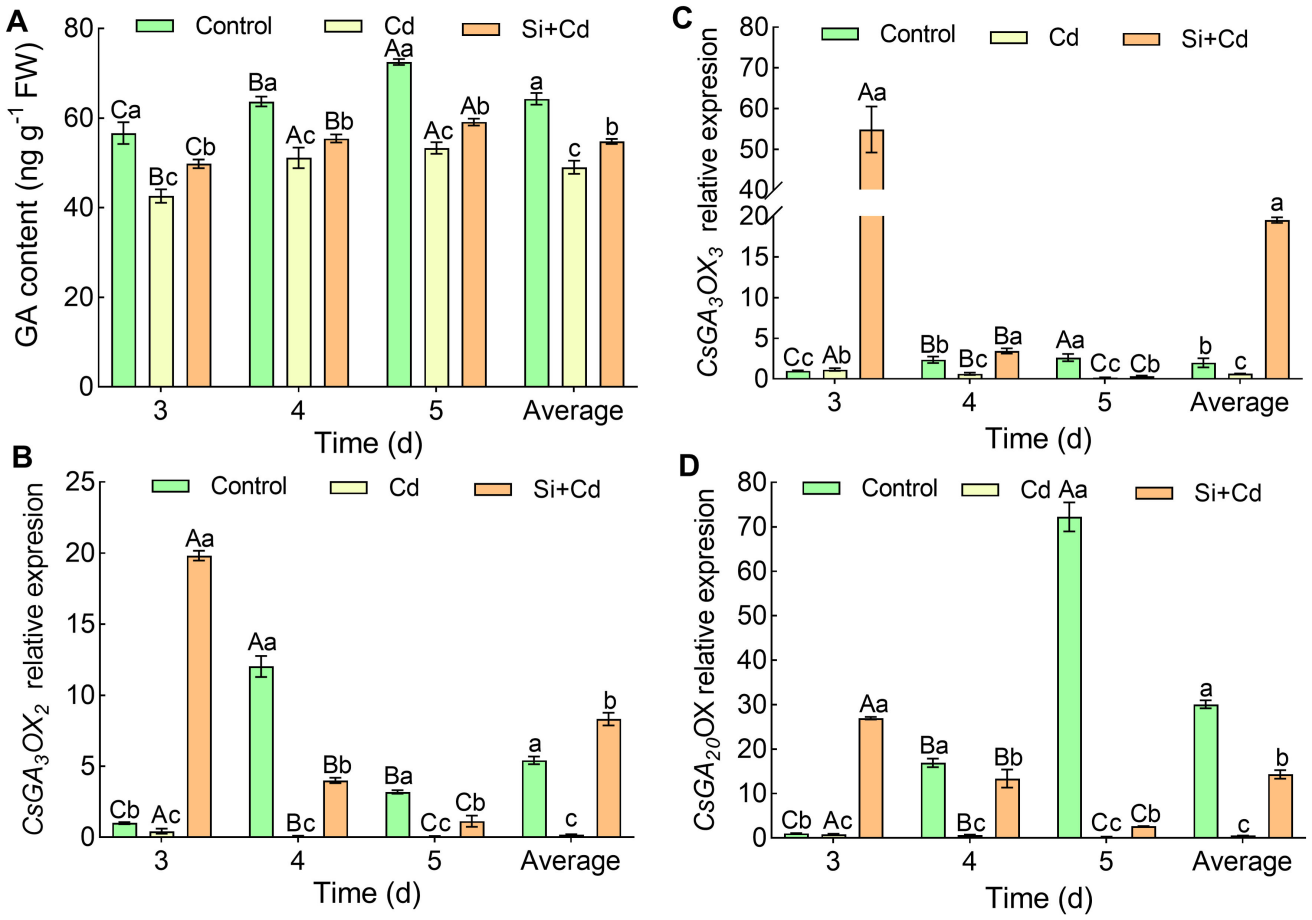
GA content **(A)**, transcript levels of *CsGA_3_OX_2_* **(B)**, *CsGA_3_OX_3_* **(C)**, *and CsGA_20_OX* **(D)** in cucumber seeds under control, Cd (150 mg/L Cd) and Cd + Si (2 mM Si + 150 mg/L Cd) treatments at 3, 4, 5 d and the average of these three days (Average). The Data are presented as mean ± SE (n = 6). Different uppercase letters indicate significant differences among time points within the same treatment. Different lowercase letters indicate significant differences among treatments at the same time point (*p* < 0.05). FW, fresh weight.

Si addition significantly reduced the increased ABA content and the relative expression levels of ABA biosynthesis-related genes, *CsNCED1* and *CsNCED2* resulted by Cd treatment (**Figures 5A–C**). The expression level of the ABA catabolism-related gene *CsCYP707A1* was enhanced (**Figure 5D**). The dramatically decreased GA contents as well as the relative expression levels of GA biosynthesis-related genes *CsGA_20_OX*, *CsGA_3_OX_2_*, and *CsGA_3_OX_3_* under Cd treatment weresignificantly increased upon Si treatment. Notably, compared to Cd treatment, the expression levels of *CsGA_20_OX*, *CsGA_3_OX_2_*, and *CsGA_3_OX_3_* with the addition of Si were 33.25, 46.09 and 47.38-fold higher on day 3, respectively (**Figure 6**). No significant interactions between treatment and time were detected for ABA and GA contents. However, significant treatment × time interactions for transcript levels of *CsNCED1*, *CsNCED2*, and *CsCYP707A1* were observed (**Supplementary Table 3**).

### 3.6 Effects of ABA and GA inhibitors and H_2_O_2_ scavenger on root length under Cd Stress

**Figure 7:**
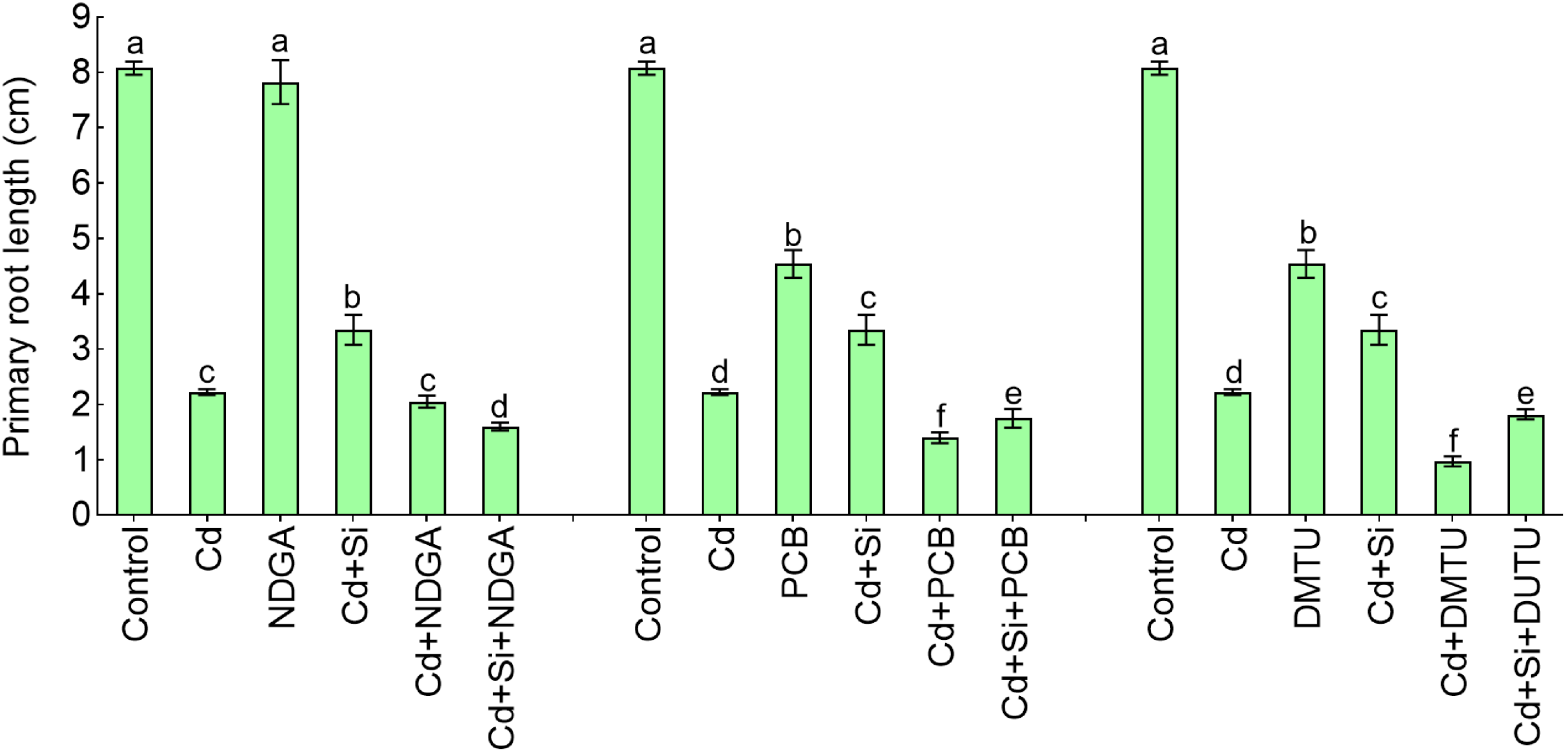
Primary root length of cucumber seeds under different treatments at day 7. Treatments included control, Cd (150 mg·kg^-^¹), Cd (150 mg·kg^-^¹) + Si (2 mM), and various inhibitors or a scavenger (NDGA, PCB, DMTU), as well as their combined treatments with Cd and/or Si. The Data are presented as mean ± SE (n = 10). Different lowercase letters indicate significant differences among treatments (*p < 0.05*).

To further test that Si promotes post-germination seedling growth under Cd stress by enhancing GA production, reducing ABA content, and increasing antioxidant enzyme activity, inhibitors or scavenger were applied during the germination of cucumber seeds. Compared to controls, treatments of Cd, PCB or DMTU, as well as Cd with either NDGA (ABA biosynthesis inhibitor), PCB (GA biosynthesis inhibitor), or DMTU (H_2_O_2_ scavenger) caused significantly decreased root length (**Figure 7**). Compared to Cd+PCB and Cd+DMTU treatments, addition of Si significantly improved primary root length by 25% and 87.63%, respectively. Similarly, primary root length was markedly reduced under Cd treatment, DMTU (H_2_O_2_ scavenger) treatment, and their combination. NDGA alone did not have significant effect on primary root length. Si addition improved the inhibited root length under Cd treatment, whereas resulted in significant lower primary root length by 21.95% compared to the Cd+NDGA treatment.

## 4 Discussion

Successful seed germination is the first step in the life cycle of plants. However, this complex process involves several signals and is influenced by both intrinsic and extrinsic factors (Miransari and Smith, 2014). A lack of uniform seed germination and consequently poor seedlings can affect both the fruit quality and crop yield. Therefore, improving the germination rate and achieving vigorous seedlings is of significant importance for agriculture. The present research investigated the capacity of Si to counteract Cd toxicity during critical phases of cucumber seed germination and early radicle elongation, as well as the underlying mechanisms involved.

### 4.1 Si improved morphological traits of cucumber seedlings under Cd stress

Roots, as the primary interface for heavy metal uptake and accumulation, play a crucial role in plant metal tolerance. Apoplastic barriers including the endodermis, exodermis, and epiblema can substantially restrict the entry and translocation of heavy metals. Inhibitory effects of Cd on growth, including suppressed cell division and elongation, have been documented in many other species, such as *Solanum lycopersicum* L (Hashem et al., 2016), *Triticum aestivum* (Ur), *Oryza sativa* (Li et al., 2020), *Hordeum vulgare* (Juknys et al., 2012), and *Helianthus annuus* (Abd_Allah et al., 2015). Our present study demonstrated that Cd treatment had no significant effects on germination rate, but notably reduced key growth metrics of germinated cucumber seeds, such as radicle length and fresh root weight (**Figures 1B, C**). We chose a Cd concentration of 150 mg/L for our subsequent experiments because this was the lowest concentration in the present study that resulted in a significant decline in both primary root length and fresh weight. Although higher concentrations resulted further declined root length and fresh weight, it is hypothesized that this sub-lethal concentration may facilitate a reliable assessment of Si-mediated alleviation, as has been adopted in other studies (Wang et al., 2025b). The insensitivity of germination rate to Cd stress observed in our study is consistent with recent reports in wheat (Han et al., 2023) and zucchini (Acila et al., 2024), where germination percentage remained unchanged while root growth was significantly inhibited under Cd exposure. A comprehensive review of Carvalho et al. (2023) also concluded that Cd effects on germination and seedling vigor can be neutral or positive, whereas post-germinative growth is more sensitive.

The present work shows that Si application significantly reversed the decline in radicle length, root and shoot weight of cucumber seedlings caused by Cd stress (**Figure 2**). Comparable alleviative effects of Si have been observed in wheat (*Triticum aestivum* L.) under Cd exposure (Ur Rahman et al., 2021). Previous studies across multiple species have demonstrated that Si application can effectively mitigate heavy metal–induced damage (Alzahrani et al., 2018; Howladar et al., 2018; Kamran et al., 2020; Shafeeq ur et al., 2020; Wan et al., 2016). It has been proposed that the non-corrosive and quasi-essential element Si fortifies these apoplastic barriers, thereby reducing Cd uptake and alleviating its phytotoxicity (Ur Rahman et al., 2021). This points to an additional, physically oriented mechanism of Si-mediated Cd detoxification, where by Si reinforces structural barriers in the root. In addition to physical barriers, Si is involved in the regulation of reactive oxygen species and the biosynthesis of phytohormones and compatible solutes, both critical processes in seed germination (Al Murad et al., 2020).

### 4.2 Si alleviates the inhibitory effects of Cd by modulating antioxidant defense and osmotic regulation

Plants activate multiple defense mechanisms to alleviate Cd toxicity, among them the antioxidant system and osmotic regulating substances play a pivotal role in controlling ROS level under Cd stress (Song et al., 2025). Hydrogen peroxide (H_2_O_2_) plays a critical role in modulating numerous physio-biochemical processes in plants (Huang et al., 2019). The detrimental effects of ROS manifest in multiple ways: morphologically, through the inhibition of root and shoot growth; and physio-biochemically, via membrane impairment and disruption of carbohydrate synthesis (Huang et al., 2019; Riaz et al., 2020). Overproduction of H_2_O_2_ inhibited plant growth and development by inducing oxidative burst. Moreover, elevated H_2_O_2_ levels can promote cell wall loosening through the oxidation of cell wall polysaccharides, ultimately resulting in cell wall degradation (Loix et al., 2017; Sabir et al., 2020). Therefore, H_2_O_2_ must be maintained at a steady-state level (Khorobrykh et al., 2020). Plants employ a suite of enzymatic antioxidants including catalase (CAT), superoxide dismutase (SOD), and peroxidase (POD), which constitute the primary defense line against oxidative stress (Hasanuzzaman et al., 2020). Specifically, SOD converts superoxide radicals (O_2_^-^) into H_2_O_2_, while CAT and POD subsequently decompose H_2_O_2_ (Ali et al., 2019). In the present study, exposure to 150 mg/L Cd triggered excessive accumulation of H_2_O_2_ and a marked reduction in soluble sugar content in cucumber radicles (**Figure 3**). Meanwhile, activities of CAT, POD, and SOD, along with the relative transcript levels of *CsSOD_(Cu-Zn)_*, *CsPOD-2-4*, and *CsCAT* were significantly upregulated under Cd treatment (**Figure 4**). These reflect the endogenous defenses of plant seeds (Song et al., 2025).

In the present study, the dramatically increased H_2_O_2_ levels upon Cd treatment were significantly declined when 2 mM Si was applied (**Figure 3A**). This is most likely due to the enhanced enzyme activities their as well as their transcript levels (**Figures 4A–C**). Particularly, POD activity shown the most pronounced increase (**Figure 4B**), which contributes to heavy metal stress tolerance not only by metabolizing H_2_O_2_ but also through participation in lignin biosynthesis, thereby reinforcing physiological barriers (Khalilzadeh et al., 2020; Riaz et al., 2021). The role of Si in modulating enzymatic antioxidant systems is further corroborated by adding the ROS scavenger of DMTU. The addition of 2 mM Si to ROS scavenger DMTU still resulted in greater radicle length under Cd stress (**Figure 7**). Thus, our results highlighted that exogenous Si application can alleviate the inhibitory effects of Cd by promoting efficient ROS-scavenging metabolic pathways (**Figure 3A**, **Figure 4**).

Soluble sugars and their derivatives are the main constituents of most geophytic storage organs. They are not only carbon sources, but also involved in osmatic regulation and in several plant developmental processes, including vegetative dormancy and growth as signaling molecules (Aguirre et al., 2018; Sheikh et al., 2022). Similar to the decrease in soluble sugar content in the embryonic axes of quinoa (*Chenopodium quinoa* Willd.) during early germination under NaCl treatment (Prado et al., 2000), we also observed that Cd resulted in lower concentrations of soluble sugars in the radicles than in the control group. Addition of Si significantly improved the soluble sugar concentration under Cd stress (**Figure 3A**). Increase in soluble carbohydrate content has been associated with adaptation to many other abiotic stresses in plant kingdom (Du et al., 2024).

### 4.3 Si regulates hormonal crosstalk to promote germination under Cd stress

Plant hormones especially ABA and GA play antagonistic roles in seed germination. ABA induces and maintains seed dormancy while inhibiting plant growth under stress conditions (Kumar et al., 2013), whereas GA promotes seed germination and subsequent growth (Abido et al., 2018). An increased GA/ABA ratio is known to enhance germination and seedling growth (El-Araby et al., 2006; Nakaune et al., 2012; Sheteiwy et al., 2017; Lemmens et al., 2019). In the present study, Si supplementation significantly reduced ABA content but increased GA levels in germinated cucumber seeds. This hormonal shift corresponded with improved radicle growth under Cd stress (**Figures 2**, **5**). The reduced ABA contents upon Si application can be attributed to decreased ABA biosynthesis together with stimulated ABA catabolism, as indicated by the downregulated transcript level of *CsNCED1* and upregulated transcript level of *CsCYP707A1*, respectively (**Figures 5C, D**) (Zhu et al., 2021). Furthermore, genes associated with GA biosynthesis, *CsGA_2_0OX*, *CsGA_3_OX_2_*, and *CsGA_3_OX_3_* were upregulated when Si is added (**Figures 5D**, **6B**). This pattern aligns with previous reports where elevated ABA levels and upregulated *CsCYP707A1* expression were observed in heat-stressed cucumber plants (Yu et al., 2018) and in proanthocyanidin-pretreated seeds and seedlings (Zhu et al., 2016, 2017). The role of Si in modulating these hormonal pathways is further strengthened by the addition of GA biosynthesis inhibitor PCB (Nicolás et al., 1996). Exogenous Si significantly increased primary root length suppressed by Cd and PCB treatment (**Figure 7**). ABA biosynthesis inhibitor NDGA had no significant effects on primary root length, which may suggest that the balance between ABA biosynthesis and catabolism was more important for seed germination and growth. Collectively, these findings suggest that Si can promote radicle growth under Cd stress partly through stimulating GA level.

## 5 Conclusions

Heavy metal contamination has become a matter of significant environmental and agricultural concern, which has a detrimental effect on plant life, resulting in a decline in crop yield and potential health risks to humans. The ability to mitigate the adverse effects of heavy metals on crop development is of paramount importance in the context of agricultural practice. In the present study, we revealed for the first time that exogenous Si application significantly alleviated radicle growth of cucumber seeds suppressed by Cd. Addition of Si helped to control ROS levels through elevated antioxidant defense system, mediating ABA/GA homeostasis, which are pivotal process involved in seed germination and early seedling development. The present study highlights the beneficial effects of exogenous Si supplementation in alleviating Cd toxicity on post-germinative seedling growth.

It is acknowledged that the sodium introduced with Na_2_SiO_3_, in conjunction with the slight pH disparity between treatment solutions and distilled water, may also have an impact on seed germination and development (Alzahrani et al., 2018; Howladar et al., 2018; Lyu et al., 2022). Nevertheless, this would not compromise the current findings on the potential of Si in counteracting Cd-induced inhibitory effects on seed germination and early development. The present study demonstrated the protective effect of Si against acute Cd exposure (Acila et al., 2024; Krishna et al., 2025) during the early post-germinative growth phase under laboratory conditions. Further studies are recommended to assess the long-term impact on plant growth and productivity in field conditions.

## Data Availability

The original contributions presented in the study are included in the article/**Supplementary Material**. Further inquiries can be directed to the corresponding author.

## Supplementary material

Supplementary Table 1 Sequences of primers used in quantitative real time PCR (qRT-PCR) experiments.
