# Correction: Exogenous silicon enhances cadmium-stressed cucumber seedling establishment by modulating antioxidant and hormonal pathways

**DOI:** 10.3389/fpls.2026.1939206

**Published:** 2026-07-23

**Authors:** Lijun Zhu, Fengli Yang, Yanping Mao, Hongying Xie, Baoguo Du

**Affiliations:** 1 School of Life Sciences (School of Ecological Forestry), Mianyang Teachers’ College, Mianyang, China; 2 Forest Ecology and Conservation in the Upper Reaches of the Yangtze River Key Laboratory of Sichuan Province, Mianyang, China; 3 Sichuan Jiuzhai Valley National Ecological Quality Comprehensive Monitoring Station, Mianyang, China; 4 Key Laboratory of Research and Conservation of Biological Diversity in Minshan Mountain of National Park of Giant Pandas at Mianyang Teachers’ College of Sichuan Provincial Department of Education, Mianyang, China

**Keywords:** abscisic acid, antioxidant system, cucumber, gibberellin, heavy metal contamination, hormone regulation, seed germination, silicon

Affiliation 3 for author Baoguo Du has been updated to “Sichuan Jiuzhai Valley National Ecological Quality Comprehensive Monitoring Station, Mianyang, China”

Affiliation 4 for author Baoguo Du has been updated to “Key Laboratory of Research and Conservation of Biological Diversity in Minshan Mountain of National Park of Giant Pandas at Mianyang Teachers’ College of Sichuan Provincial Department of Education”

The original version of this article has been updated.

