# Supplementary material for: Exogenous silicon enhances cadmium-stressed cucumber seedling establishment by modulating antioxidant and hormonal pathways
Source: Front Plant Sci. 2026 Jul 13;17:1851047. doi: 10.3389/fpls.2026.1851047 (PMC13444270; doi:10.3389/fpls.2026.1851047)
Supplement: Supplementary file 1 [file Table1.doc]

**Supplementary materials**

Table S1. Sequences of primers used in quantitative real time PCR (qRT-PCR) experiments

**Gene	Accession number	Forward primer	Reverse primer**

*CsSOD _(Cu-Zn)_*	101231664	GAGCAATCAGGGAGTCAGT	TACCATCATCACCAGCAAC

*CsPOD-2-4*	101228793	TTCTTGCCCTTCAGGTTGT	CTCCGATTGATTTGTTCCA

*CsCAT*	Csa6M356990	AACAACACCGCCGTAATGT	ATGACGGGGGTTTGGACG

*CsGA_20_OX*	Csa3M179110	ATCCGTTCCTTATGTTGCTG	CCTCATTATTGATTCATTGTCC

*CsGA_3_OX_2_*	101222627	CTTTAGCGGAGCAAATCAC	CGTAGAAATAAGCCATAGACAA

*CsGA_3_OX_3_*	101219147	AAGAGTCTATGTGGAAGGGTG	CCGTGGGATAAAGTAATGG

*CsCYP707A1* Csa4M056600.1 TCGGAGTTCTGTTTGCGGCT	TGGTAAAGGGCATAGTTCGT

*CsNCED1*	Csa4M064690.1	CAGGGGGTTATTTGGTCTTGTT	ATCATCGTTGGCTGAGGCA

*CsNCED2*	Csa7M428120.2	CAAATCCGAAGTTTAGCCCAG	CATAATCCAGCAGACCAAGCG

*Actin	DQ641117	AGAGATGGCTGGAATAGAAC	CTGGTGATGGTGTGAGTC CsACS2*

TABLES2 Results of two‑way ANOVA for the effects of treatment and time on H_2_O_2_ and soluble sugar contents, antioxidant enzyme activities (SOD, POD, CAT), and transcript levels of *CsSOD(Cu–Zn)*, *CsPOD‑2‑4*, and *CsCAT* in cucumber seeds under Cd stress.

| Parameters | df | H_2_O_2_content | | Soluble sugar | | SOD | | POD | | CAT | | *CsSOD_(Cu–Zn)_* | | *CsPOD-2–4* | | *CsCAT* | |
| --- | --- | --- | --- | --- | --- | --- | --- | --- | --- | --- | --- | --- | --- | --- | --- | --- | --- |
|  |  | *F* | *p* | *F* | *p* | *F* | *p* | *F* | *p* | *F* | *p* | *F* | *p* | *F* | *p* | *F* | *p* |
| Treatment | 3 | 75.32 | <0.0001 | 13.64 | 0.0002 | 44.07 | <0.0001 | 72.56 | <0.0001 | 18.31 | <0.0001 | 25.49 | <0.0001 | 15.97 | 0.0001 | 16.43 | <0.0001 |
| Time | 3 | 61.65 | <0.0001 | 42.87 | <0.0001 | 8.599 | 0.0007 | 10.31 | 0.0002 | 2.964 | 0.0681 | 15.57 | <0.0001 | 18.98 | <0.0001 | 16.36 | <0.0001 |
| Treatment×Time | 9 | 57.97 | <0.0001 | 1.852 | 0.1628 | 0.3980 | 0.8090 | 24.96 | <0.0001 | 24.49 | <0.0001 | 14.33 | <0.0001 | 9.816 | 0.0002 | 4.109 | 0.0154 |

TABLE S3 Results of two‑way ANOVA for the effects of treatment and time on ABA and GA contents, and transcript levels of ABA biosynthesis genes (*CsNCED1*, *CsNCED2*), ABA catabolism gene (*CsCYP707A1*), and GA biosynthesis genes (*CsGA_3_OX_2_*, *CsGA_3_OX_3_*, *CsGA_20_OX*) in cucumber seeds under Cd stress.

| Parameters | df | ABA | | GA | | *CsNCED1* | | *CsNCED2* | | *CsCYP707A1* | | *CsGA_3_OX_2_* | | *CsGA_3_OX_3_* | | *CsGA_20_OX* | |
| --- | --- | --- | --- | --- | --- | --- | --- | --- | --- | --- | --- | --- | --- | --- | --- | --- | --- |
|  |  | *F* | *p* | *F* | *p* | *F* | *p* | *F* | *p* | *F* | *p* | *F* | *p* | *F* | *p* | *F* | *p* |
| Treatment | 3 | 3.673 | 0.0460 | 7.434 | 0.0044 | 70.54 | <0.0001 | 23.75 | <0.0001 | 39.13 | <0.0001 | 59.02 | <0.0001 | 9.106 | 0.0019 | 7.891 | 0.0035 |
| Time | 3 | 3.258 | 0.0620 | 3.658 | 0.0465 | 69.38 | <0.0001 | 39.26 | <0.0001 | 69.25 | <0.0001 | 73.05 | <0.0001 | 10.03 | 0.0012 | 22.62 | <0.0001 |
| Treatment×Time | 9 | 0.4539 | 0.7683 | 0.2667 | 0.8955 | 28.25 | <0.0001 | 12.95 | <0.0001 | 43.93 | <0.0001 | 62.00 | <0.0001 | 9.496 | 0.0003 | 22.83 | <0.0001 |
